# Factors affecting the willingness of nursing care staffs for cooperation with heart failure care and the role of internet video education

**DOI:** 10.1002/jgf2.658

**Published:** 2023-11-20

**Authors:** Yoshiharu Kinugasa, Toshiaki Adachi, Masaharu Fukuki, Yutaka Hirota, Natsuko Ishiga, Masahiko Kato, Einosuke Mizuta, Emiko Mura, Yoshihito Nozaka, Hiroki Omodani, Hiroaki Tanaka, Yasunori Tanaka, Izuru Watanabe, Kazuhiro Yamamoto, Masaaki Mikami

**Affiliations:** ^1^ Department of Cardiovascular Medicine and Endocrinology and Metabolism, Faculty of Medicine Tottori University Yonago Japan; ^2^ Adachi Clinic Yonago Japan; ^3^ Department of Cardiology Yonago Medical Center Yonago Japan; ^4^ Tomimasu Surgical Primary Care Clinic Yonago Japan; ^5^ Division of Rehabilitation Tottori University Hospital Yonago Japan; ^6^ Department of Pathobiological Science and Technology, School of Health Science, Faculty of Medicine Tottori University Yonago Japan; ^7^ Department of Cardiology Sanin Rosai Hospital Yonago Japan; ^8^ Visiting Nurse Station Nanbu Kohoen Yonago Japan; ^9^ Nozaka Clinic Yonago Japan; ^10^ Omodani Internal Medicine and Cardiovascular Medicine Clinic Yonago Japan; ^11^ Department of Cardiology Tottori Prefecture Sakaiminato General Hospital Sakaiminato Japan; ^12^ Department of Cardiology Hakuai Hospital Yonago Japan; ^13^ Department of Nursing Sanin Rosai Hospital Yonago Japan; ^14^ Hoshoji Clinic Nanbucho Japan

**Keywords:** collaborative care, education, internet

## Abstract

**Background:**

With the aging of heart failure (HF) patients, collaboration between medical and nursing care facilities is essential for HF care. The aims of this study were: (1) to identify the factors that affect willingness of nursing care staffs to cooperate with HF care; (2) to test whether the internet video education is useful in improving their willingness to collaborate.

**Methods:**

A web‐based questionnaire was e‐mailed to 417 registered medical corporations that operated nursing care facilities in the prefecture where the authors work. Medical and care staff working at each facility were asked their willingness to cooperate with HF care and their problems about collaboration. Machine learning analysis was used to assess the factors associated with unwillingness to cooperate. After watching a 6‐min YouTube video explaining HF and community collaboration, we reaffirmed their willingness to cooperate.

**Results:**

We received responses from 76 medical and care staff members. Before watching the video, 32.9% of participants stated that they were unwilling to cooperate with HF care. Machine learning analysis showed that job types, perceived problems of collaboration, and low opportunities to learn about HF were associated with unwillingness to cooperation. After watching the video, we observed an increase from 67.1% to 80.3% (*p* < 0.05) of participants willing to cooperate with HF care.

**Conclusions:**

Job types, perceived problems of collaboration, and low opportunities to learn about HF are associated with unwillingness of nursing care staff for HF care. Internet videos are potential learning tool that can easily promote community collaboration for HF.

## Graphical abstract

Factors associated with unwillingness to cooperate with heart failure care.

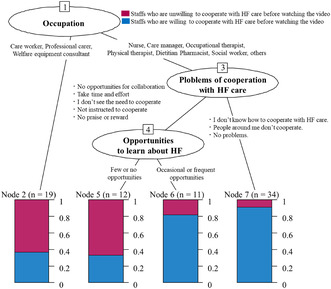

## 1 INTRODUCTION

In recent years, patients with heart failure (HF) have become older.^1^ Elderly patients have difficulty practicing self‐care management for HF owing to the high frequency of comorbid psychological frailty (cognitive decline). In addition, the lack of caregivers because of social frailty (reduced social connections) increases the risk of HF exacerbation.^2^ One intervention for frail HF patients is employing social welfare resources.^3^, ^4^, ^5^, ^6^ Interventions using home‐care services (including home nursing care) reportedly reduce HF exacerbation and improve quality of life.^7^ Thus, there is an increasing need for collaboration between medical institution and local nursing care facilities.

Increasing the willingness of medical and care staffs to collaborate with HF care is dispensable to promote local collaboration. However, the degree of their willingness to collaborate with HF care and the factors that influence their willingness have not been examined. In addition, the lack of knowledge of HF care among medical and care staffs is one of the barriers to collaboration, and the need for education for them has been proposed.^8^ However, it is unclear whether staff education promotes their willingness to collaborate with HF care. Thus, the aims of this study were: (1) to identify the degree of willingness of staffs working at nursing care facilities to cooperate with HF care and the factors that affect their willingness; (2) to test whether staff education using the Internet video, which have been widely used as educational tools for health‐care professionals and HF patients, is useful in improving willingness to collaborate.

## 2 METHODS

### 2.1 Questionnaire survey

We e‐mailed a web‐based questionnaire to representatives of 417 registered medical corporations that operated nursing care facilities in the prefecture where author works in Japan, and responses were received from September 14th to October 7th, 2022. The web‐based questionnaire was conducted using a google form. A link to the YouTube video was inserted in the google form, and respondents can watch the YouTube video directly from the questionnaire. Medical and care staff working at each facility were asked their willingness to cooperate with HF care and their problems about collaboration. After watching a 6‐min educational video, which explained HF and community collaboration for HF, we reaffirmed their willingness to cooperate. The questionnaire content and educational video appear in Table S1 and Figure S1. We did not know how many staff members at each institution were asked to respond to the questionnaire, so we were unable to calculate the response rate. We received 78 responses; two were from clerical staff, we excluded them from analysis, leaving 76 participants. In the questionnaire, we asked, “What do you think about working together in the community to prevent HF?” Participants were asked to select one of the following five responses: (1) I am not interested in cooperating with HF care; (2) I'm interested in cooperating with HF care but unwilling to do so; (3) I am interested in cooperating with HF care and want to do so; (4) I'm now cooperating with HF care, but I'm not confident about continuing to do so; (5) I'm now cooperating with HF care and confident about continuing. We defined participants who responded (1) or (2) as staff who were unwilling to cooperate with HF care; we categorized participants who responded (3), (4), or (5) as staff who were willing to cooperate with HF care.

### 2.2 Educational video

**FIGURE 1:**
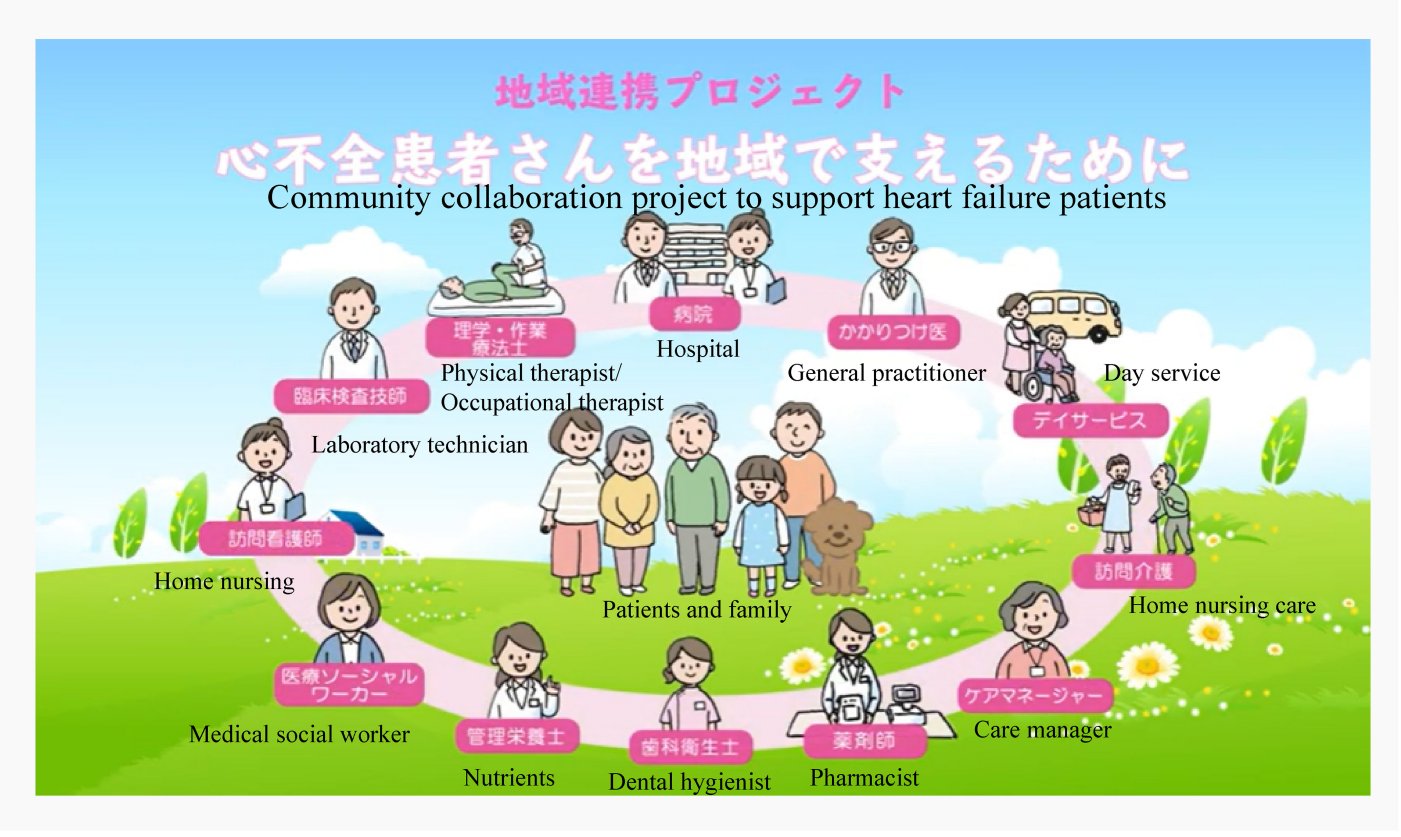
Educational video used in this study. The scenarios for each video are described in Figures S1–S3. HF, heart failure.

The medical association in the prefecture where authors work has produced a video to promote regional cooperation in HF (Figure 1). This video explains HF and community collaboration with HF. The 6‐min video comprises three parts. Part 1 explains the significance of community collaboration in HF care. Part 2 clarifies key points to observe in HF patients, such as weight gain, worsening edema, and worsening shortness of breath. Part 3 details how to use the HF handbook. The scenarios for each part of the video appear in Figures S1–S3 (the video can be viewed on YouTube).

### 2.3 Statistical analysis

We used Fisher's analysis to compare categorical variables between the two participant groups. Multiple comparisons between pairs were assessed using the Bonferroni test. We made paired comparisons of the proportions of the two groups before and after watching videos using the McNemar's test. To assess the factors associated with respondents who were unwilling to cooperate with HF care, we conducted classification and regression tree (CART) analysis. CART is a predictive algorithm used in machine learning; it describes how the objective variable is predicted based on other variables. An optimal predictive variable is selected and split at an appropriate cutoff value. We entered the following factors into the analysis: age; gender; occupation, workplace; opportunities to learn about HF; problems in collaboration with HF care; understanding the content of the videos; and evaluation of the videos. We considered *p* < 0.05 statistically significant. All analyses were performed using R version 4.1.2 (R Foundation for Statistical Computing, Vienna, Austria).

## 3 RESULTS

Table 1 shows the demographics of the 76 participants. Most were in their 40s, followed by ones in their 50s. Approximately, 70% were women. The most common occupations were nurses, followed by care managers and care workers. The most common workplace was nursing homes, followed by day care or day service.

**TABLE 1:** Questionnaire responses.

	Overall (*n* = 76)	Staffs who answered willing to cooperate with HF care before watching the videos (*n* = 51)	Staffs who answered not willing to cooperate with HF care before watching the videos (*n* = 25)	*p* value
Age, *n* (%)	20–29 y/o: 4 (5.3)	20–29 y/o: 2 (3.9)	20–29 y/o: 2 (8.0)	0.839
	30–39 y/o: 15 (19.7)	30–39 y/o: 11 (21.6)	30–39 y/o: 4 (16.0)	
	40–49 y/o: 27 (35.5)	40–49 y/o: 19 (37.3)	40–49 y/o: 8 (32.0)	
	50–59 y/o: 18 (23.7)	50–59 y/o: 12 (23.5)	50–59 y/o: 6 (24.0)	
	60 or more y/o: 12 (15.8)	60 or more y/o: 7 (13.7)	60 or more y/o: 5 (20.0)	
Female, *n* (%)	55 (72.4)	39 (76.5)	16 (64.0)	0.284
Occupation, *n* (%)	Nurse: 24 (31.6)	Nurse: 19 (37.3)	Nurse: 5 (20.0)	0.109
	Care manager: 14 (18.4)	Care manager: 9 (17.6)	Care manager: 5 (20.0)	
	Care worker: 14 (18.4)	Care worker: 6 (11.8)	Care worker: 8 (32.0)	
	Occupational therapist: 5 (6.6)	Occupational therapist: 4 (7.8)	Occupational therapist: 1 (4.0)	
	Physical therapist: 4 (5.3)	Physical therapist: 3 (5.9)	Physical therapist: 1 (4.0)	
	Dietitian: 3 (3.9)	Dietitian: 2 (3.9)	Dietitian: 1 (4.0)	
	Pharmacist: 3 (3.9)	Pharmacist: 3 (5.9)	Pharmacist: 0 (0.0)	
	Welfare equipment consultant: 3 (3.9)	Welfare equipment consultant: 0 (0.0)	Welfare equipment consultant: 3 (12.0)	
	Professional carer: 2 (2.6)	Professional carer: 1 (2.0)	Professional carer: 1 (4.0)	
	Social worker: 2 (2.6)	Social worker: 2 (3.9)	Social worker: 0 (0.0)	
	Others: 2 (2.6)	Others: 2 (3.9)	Others: 0 (0.0)	
Workplace, *n* (%)	Nursing home: 26 (34.2)	Nursing home: 16 (31.4)	Nursing home: 10 (40.0)	0.570
	Day care/Day services: 15 (19.7)	Day care/Day services: 10 (19.6)	Day care/Day services: 5 (20.0)	
	Home care support services: 10 (13.2)	Home care support services: 6 (11.8)	Home care support services: 4 (16.0)	
	Home nursing station: 8 (10.5)	Home nursing station: 7 (13.7)	Home nursing station: 1 (4.0)	
	Home care station: 2 (2.6)	Home care station: 2 (3.9)	Home care station: 0 (0.0)	
	Hospital/clinic: 5 (6.6)	Hospital/clinic: 4 (7.8)	Hospital/clinic: 1 (4.0)	
	Pharmacy: 3 (3.9)	Pharmacy: 3 (5.9)	Pharmacy: 0 (0.0)	
	Others: 7 (9.2)	Others: 3 (5.9)	Others: 4 (16.0)	
Q2. Opportunities to learn about HF, *n* (%)	No opportunities at all: 8 (10.5)	No opportunities at all: 2 (3.9)*	No opportunities: 6 (24.0)*	0.004
	Few opportunities: 31 (40.8)	Few opportunities: 18 (35.3)	Few opportunities: 13 (52.0)	
	Occasional opportunities: 34 (44.7)	Occasional opportunities: 28 (54.9)*	Occasional opportunities: 6 (24.0)*	
	Frequent opportunities: 3 (3.9)	Frequent opportunities: 3 (5.9)	Frequent opportunities: 0 (0.0)	
Q3. Willingness to participate learning about HF, *n* (%)	I do not want to participate: 5 (6.6)	I do not want to participate: 3 (5.9)	I do not want to participate: 2 (8.0)	0.009
	I want to participate: 53 (69.7)	I want to participate: 31 (60.8)*	I want to participate: 22 (88.0)*	
	I want to participate very much: 18 (23.7)	I want to participate very much: 17 (33.3)*	I want to participate very much: 1 (4.0)*	
Q4. Problems of cooperation with HF care, *n* (%)	I do not know how to cooperate: 36 (47.4)	I do not know how to cooperate: 27 (52.9)	I do not know how to cooperate: 9 (36.0)	0.063
	No opportunities for collaboration: 17 (22.4)	No opportunities for collaboration: 9 (17.6)	No opportunities for collaboration: 8 (32.0)	
	There is no problem: 7 (9.2)	There is no problem: 6 (11.8)	There is no problem: 1 (4.0)	
	Take time and effort: 4 (5.3)	Take time and effort: 2 (3.9)	Take time and effort: 1 (4.0)	
	People around me do not cooperate: 4 (5.3)	People around me do not cooperate: 4 (7.8)	People around me do not cooperate: 0 (0.0)	
	I do not see the need to cooperate: 3 (3.9)	I do not see the need to cooperate: 0 (0.0)	I do not see the need to cooperate: 3 (12.0)	
	Not instructed to cooperate: 3 (3.9)	Not instructed to cooperate: 2 (3.9)	Not instructed to cooperate: 1 (4.0)	
	No praise or reward: 2 (2.6)	No praise or reward: 1 (2.0)	No praise or reward: 1 (4.0)	
Q5–7. Understanding the content of the videos, *n* (%)	Video 1: Understood: 36 (47.4)	Video 1: Understood: 21 (41.2)	Video 1: Understood: 15 (60.0)	0.147
	Well understood: 40 (52.6)	Well understood: 30 (58.8)	Well understood: 10 (40.0)	
	Video 2: Understood: 22 (28.9)	Video 2: Understood: 12 (23.5)	Video 2: Understood: 10 (40.0)	0.180
	Well understood: 54 (71.1)	Well understood: 39 (76.5)	Well understood: 15 (60.0)	
	Video 3: Understood: 26 (34.2)	Video 3: Understood: 15 (29.4)	Video 3: Understood: 11 (44.0)	0.304
	Well understood: 50 (65.8)	Well understood: 36 (70.6)	Well understood: 14 (56.0)	
Q8. Evaluation of the videos, *n* (%)	Good: 70 (92.1)	Good: 50 (98.0)*	Good: 20 (80.0)*	0.005
	Good nor bad: 4 (5.3)	Good nor bad: 0 (0.0)*	Good nor bad: 4 (16.0)*	
	Bad: 2 (2.6)	Bad: 1 (2.0)	Bad: 1 (4.0)	

*Note*: Asterixis indicates items with a significant difference (*p* < 0.05) among the groups.

Abbreviations: HF, heart failure; y/o, years old.

**FIGURE 2:**
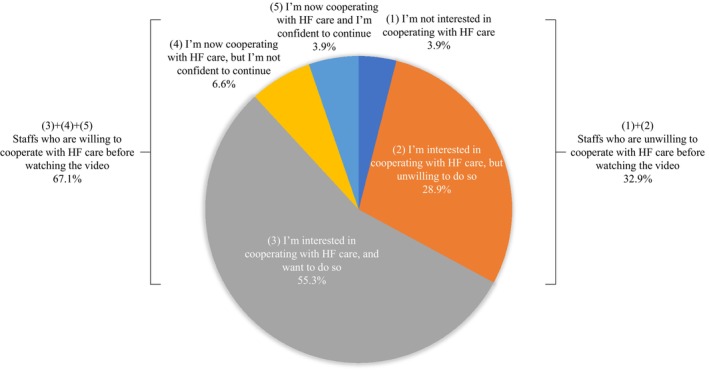
Responses on willingness to cooperate with HF care. HF, heart failure.

Figure 2 shows the responses to question 1 (“What do you think about working together in the community to prevent HF?”). Before watching the video, 11.9% of respondents stated that they were cooperating with HF care; 55.3% said they were interested in cooperating with HF care and wanted to do so. Thus, 67.1% of participants were willing to cooperate with HF care. However, 3.9% replied that they were not interested in cooperating with HF care; 28.9% were interested in cooperating with HF care but were unwilling to do so. Thus, 32.9% of respondents were unwilling to cooperate with HF care.

Responses to the other questions appear in Table 1. Regarding question 2 (“Are there opportunities to learn about HF?”), over half of participants stated that they had few or no opportunities to learn about HF. For question 3 (“Would you like to participate in any opportunities to lean about HF?”), more than 90% of respondents said that they would like to participate. With item 4 (“Please tell us about any problems with cooperation with HF care”), the most common response was that they did not know how to cooperate; that was followed by having no opportunities to cooperate. Regarding items 5–7 (“Did you understand the video content?”), all respondents said that they understood the content of parts 1–3. For question 8 (“Do you think that the video is a good way to learn about HF?”), over 90% rated it favorably.

Table 1 also shows a comparison of the two groups of participants who were willing to cooperate with HF care and those who were unwilling to do so based on the questionnaire responses before watching the video. Participants who were unwilling to cooperate with HF care had significantly fewer opportunities to learn about HF than those who were willing to do so (*p* < 0.05). The former were also less willing to participate in learning opportunities than those willing to cooperate with HF care (*p* < 0.05). We observed no differences between the two groups in understanding the video. Participants who were unwilling to cooperate with HF care rated the video lower than those who were willing to cooperate with such care. However, even among respondents who were unwilling to cooperate, 80% rated the video favorably.

**FIGURE 3:**
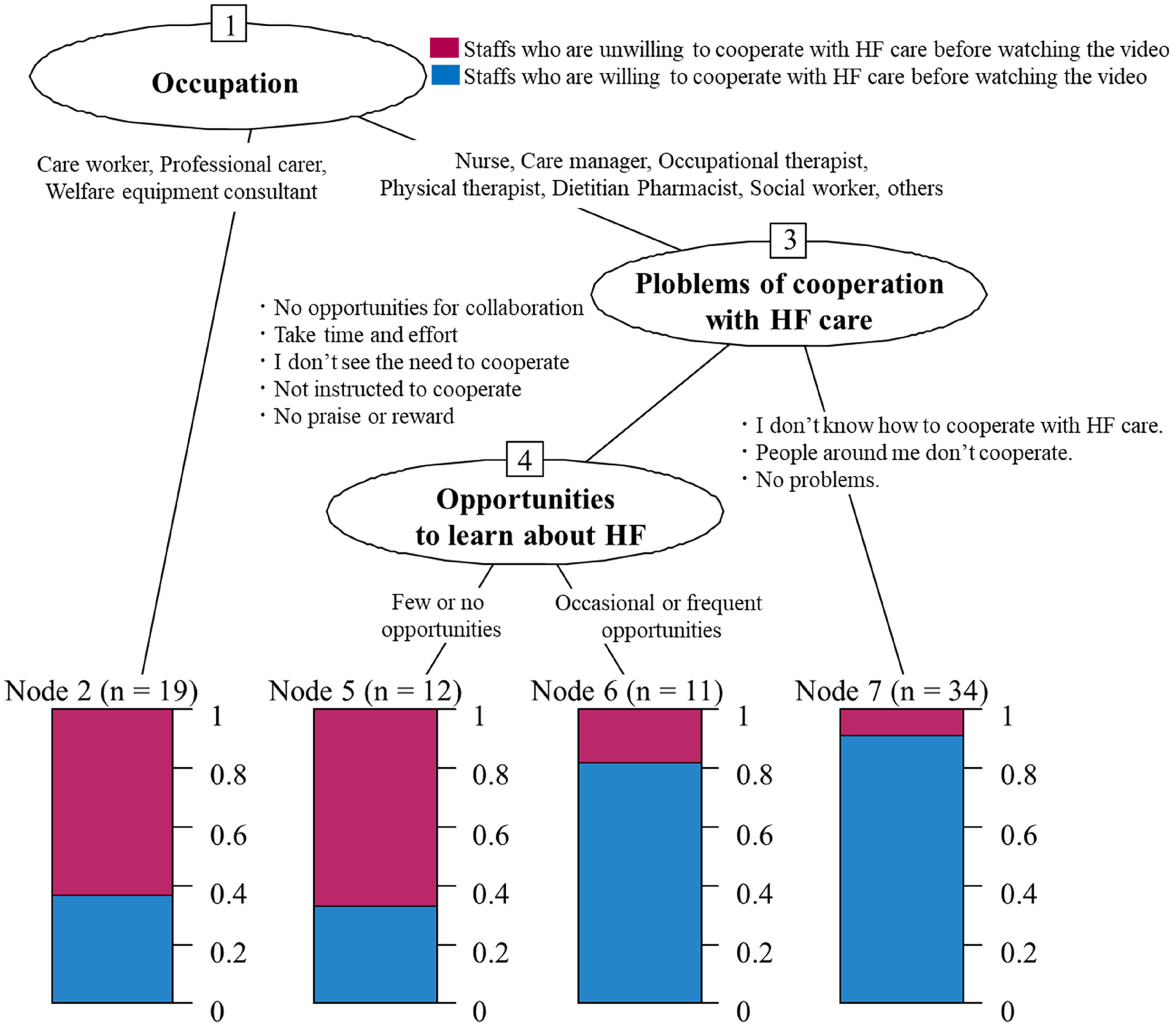
Factors associated with unwillingness to cooperate with HF care assessed using CART analysis. CART, classification and regression tree; HF, heart failure.

Figure 3 presents the factors associated with participants who were unwilling to cooperate with HF care assessed using CART analysis. In terms of occupation, care workers, professional carers, and welfare equipment consultants evidenced a lack of willingness to cooperate with HF care. With other occupations, there were differences in problems with community collaboration with HF care between participants willing and unwilling to cooperate. Participants willing to cooperate were more likely to say that they did not know how to cooperate and that the people around them did not cooperate than participants unwilling to cooperate. Conversely, participants who were unwilling to cooperate with HF care were more likely to state that they had no opportunities for cooperation and did not see the need to cooperate than participants who were willing to cooperate. In addition, the former participants had fewer opportunities to learn about HF than those willing to cooperate.

**FIGURE 4:**
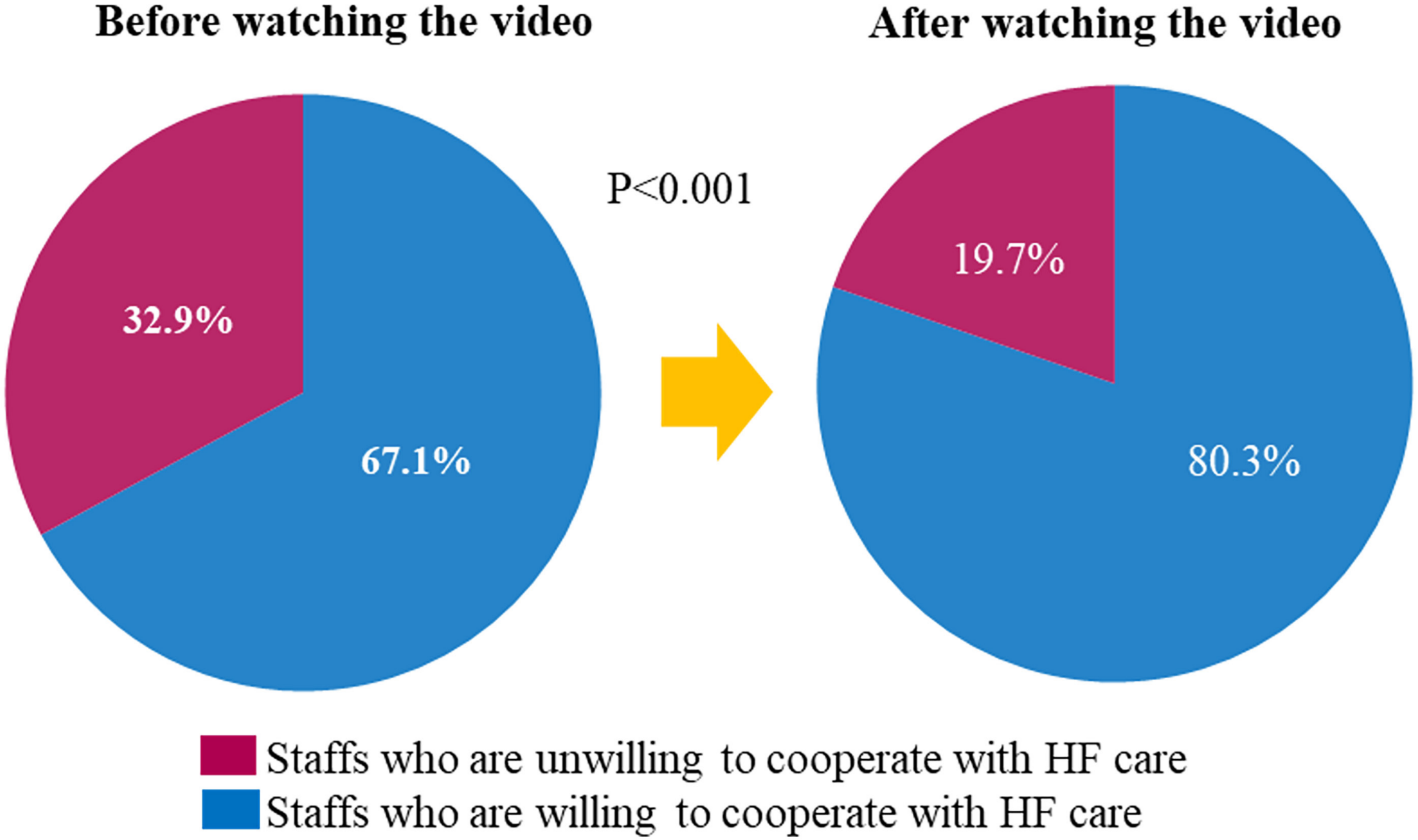
Changes in responses on willingness to cooperate with HF care before and after watching the video. HF, heart failure.

Figure 4 shows the change in the proportion of participants who were willing to cooperate with HF care before and after watching the video. There was a significant increase from 67.1% to 80.3% (*p* < 0.05) in the proportion of respondents willing to cooperate. There were no significant differences in the background information collected between the staffs who have become cooperative with HF care and those who have not.

## 4 DISCUSSION

This study showed that approximately 30% of staffs working at nursing care facilities were not cooperative in community collaboration in HF care. Factors influencing unwillingness to care were related to job type, perceived problems of collaboration, and low opportunities to learn about HF care. Internet‐based education may increase their willingness to collaborate with HF care.

This is the first report in Japan to assess the barriers of staffs working at nursing facilities to collaborate with HF care. Of these factors, providing educational opportunities to learn about HF is a factor that can be intervened. To provide effective education, it is reasonable to approach according to the transtheoretical model. The transtheoretical model is a behavior change model that comprises six stages of change (pre‐contemplation, contemplation, preparation, action, maintenance, and termination); it provides a guideline for understanding individual motivation in leading to better behavior.^9^ According to that model, participants who were unwilling to cooperate with HF care were in the pre‐contemplation phase or contemplation phase: The former had lack of interest in HF care owing to insufficient knowledge; the latter were interested but unable to change their behavior owing to barriers to action. Participants who were unwilling to cooperate with HF care had significantly fewer opportunities to learn about HF. Thus, to promote community collaboration, it is necessary to give those staff appropriate knowledge and individual motivation. Most participants unwilling to cooperate stated that the problems with regional cooperation were not understanding the significance of the cooperation, lack of opportunities, and excessive time and effort. Therefore, it is necessary to emphasize the need for collaboration and ease in taking action to produce positive behavior change.^10^ In recent years, social media, such as Internet video, have been widely used as learning tools for health‐care professionals and HF patients.^11^, ^12^ This study demonstrates the benefits of an Internet video in motivating medical and care staff to participate in community collaboration in HF. There have been no reports that Internet videos can lead to positive behavior changes among health and care professionals with HF. The video used in this study comprised three parts—each approximately 2 min long for a total of 6 min. We observed an educational effect even with such a short video. Regarding optimal duration of social media videos, it has been reported that with longer videos, there is greater likelihood of not being completely viewed.^13^ The video marketing company Wistia has reported that 2–6 min is the optimal length to sustain viewer interest.^13^ Thus, the short video we used was ideal for promoting community collaboration with HF care.

In this study, we observed differences in motivation for collaboration among the professional staff. Notably, social care workers and professional carers had low motivation for HF care. It is necessary to consider appropriate education for such professions because they are often in contact with HF patients.

This study has several limitations. The survey was not requested directly to the medical and nursing care staff, but indirectly through the representatives of the facilities. This may affect the low response rate of the questionnaire. We did not compare the educational effects of Internet videos versus other interventions such as using pamphlets or lecture, which is a limitation of this study. This study assessed the change in respondents' motivation as the educational effect. However, it was not able to assess the behavioral change after the educational intervention. There may have been a bias in that the participants were interested in HF care. That factor needs to be considered when interpreting our results. Finally, we conducted our study with a small number of participants in one region. Further research should be undertaken with a larger number of participants in a wider region.

In conclusion, job type, perceived problems of collaboration, and low opportunities to learn about HF are associated with unwillingness of medical and care staff for HF care. Thus, providing opportunities to learn about HF is important to motivate cooperation in care, and social media is a potential learning tool that can easily promote community collaboration for HF.

## FUNDING INFORMATION

This study was supported by a grant from the Japan Society for the Promotion of Science (JSPS KAKENHI Grant No. 20K08403).

## CONFLICT OF INTEREST STATEMENT

The authors have stated explicitly that there are no conflicts of interest in connection with this article.

## ETHICS STATEMENT

The investigation confirmed to the principles outlined in the Declaration of Helsinki. The questionnaire did not collect personally identifiable information; respondents could refuse to participate or decline to answer an item, and there was no psychological burden. So, it did not fall under the national ethical guidelines for clinical research. Therefore, the Institutional Review Board (IRB) of our institution approved this study without ethical review. The Institutional Review Board decided that the responses to the questionnaire could be regarded as informed consent to participate in this study and allowed that written informed consent was omitted.

## Supporting information


Appendix S1.

Table S1.

## Data Availability

Research data are not shared.
