# Supplementary material for: Factors affecting the willingness of nursing care staffs for cooperation with heart failure care and the role of internet video education
Source: J Gen Fam Med. 2023 Nov 20;25(1):19–27. doi: 10.1002/jgf2.658 (PMC10792320; doi:10.1002/jgf2.658)
Supplement: Supplementary file 1 — Appendix S1. [file JGF2-25-19-s002.pptx]

## Slide 1
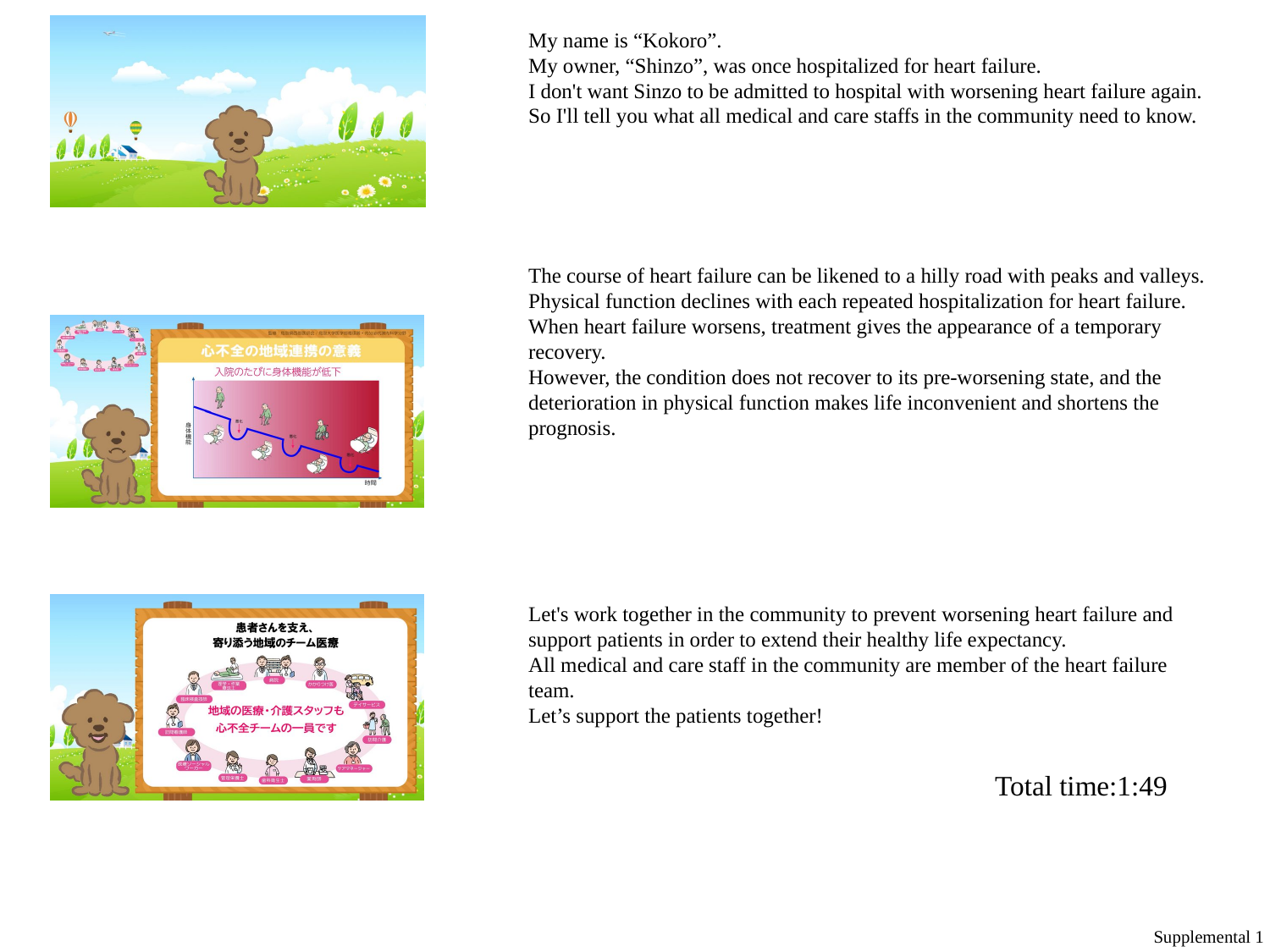

My name is “Kokoro”.
My owner, “Shinzo”, was once hospitalized for heart failure.
I don't want Sinzo to be admitted to hospital with worsening heart failure again.
So I'll tell you what all medical and care staffs in the community need to know.
The course of heart failure can be likened to a hilly road with peaks and valleys.
Physical function declines with each repeated hospitalization for heart failure. When heart failure worsens, treatment gives the appearance of a temporary recovery.
However, the condition does not recover to its pre-worsening state, and the deterioration in physical function makes life inconvenient and shortens the prognosis.
Let's work together in the community to prevent worsening heart failure and support patients in order to extend their healthy life expectancy.
All medical and care staff in the community are member of the heart failure team.
Let’s support the patients together!
Total time:1:49
Supplemental 1

## Slide 2
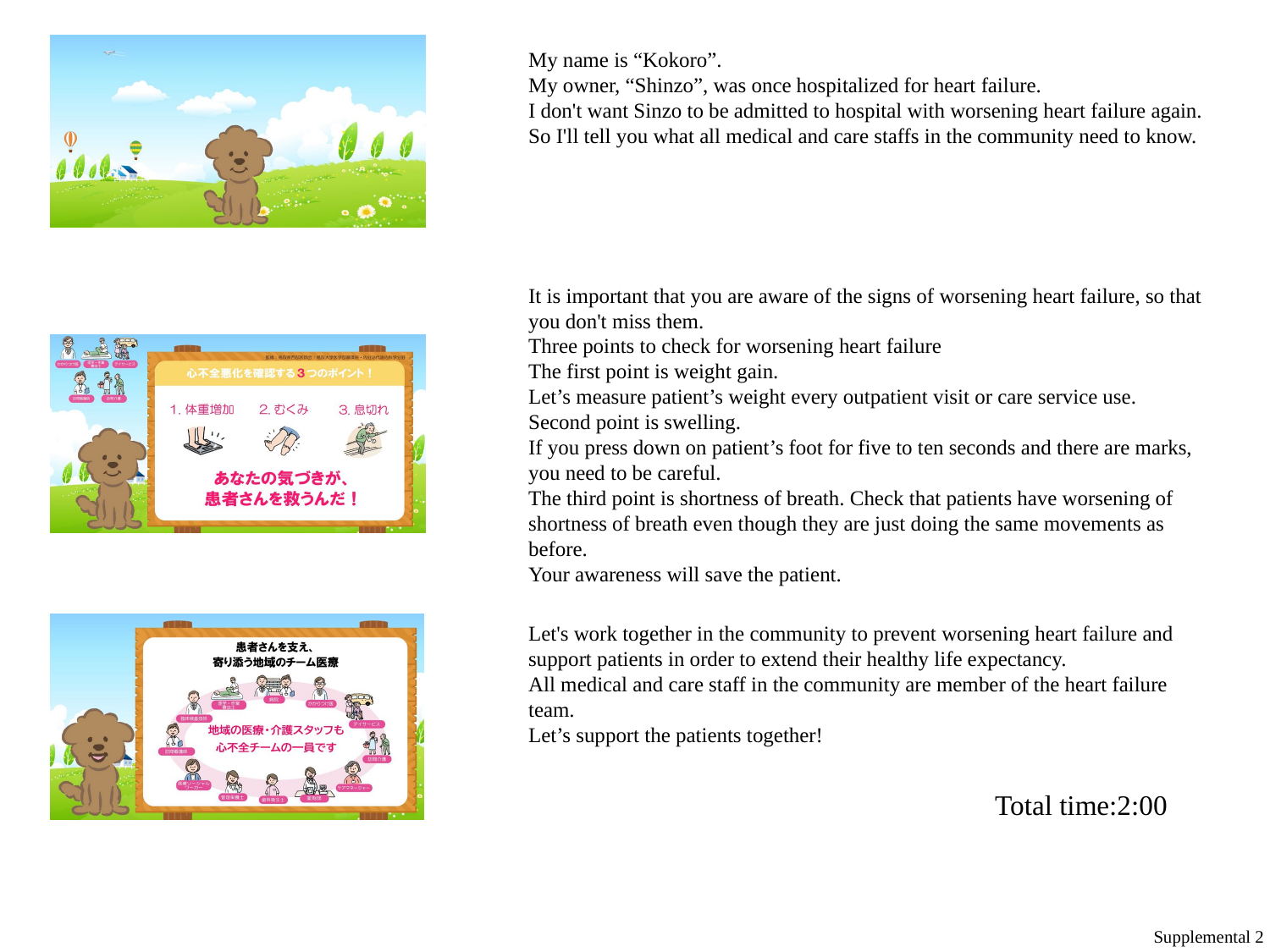

My name is “Kokoro”.
My owner, “Shinzo”, was once hospitalized for heart failure.
I don't want Sinzo to be admitted to hospital with worsening heart failure again.
So I'll tell you what all medical and care staffs in the community need to know.
It is important that you are aware of the signs of worsening heart failure, so that you don't miss them.
Three points to check for worsening heart failure
The first point is weight gain.
Let’s measure patient’s weight every outpatient visit or care service use.
Second point is swelling.
If you press down on patient’s foot for five to ten seconds and there are marks, you need to be careful.
The third point is shortness of breath. Check that patients have worsening of shortness of breath even though they are just doing the same movements as before.
Your awareness will save the patient.
Let's work together in the community to prevent worsening heart failure and support patients in order to extend their healthy life expectancy.
All medical and care staff in the community are member of the heart failure team.
Let’s support the patients together!
Total time:2:00
Supplemental 2

## Slide 3
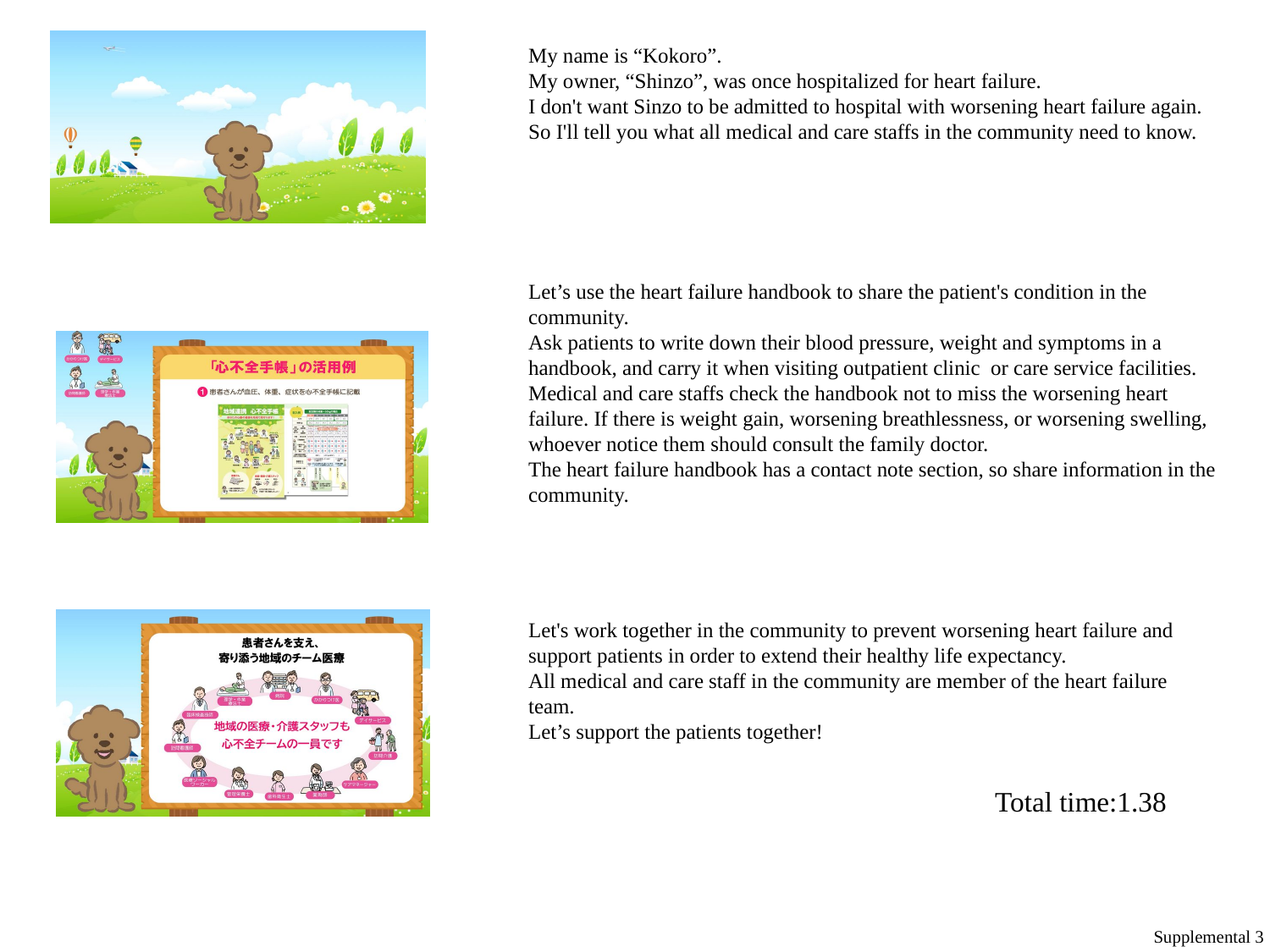

My name is “Kokoro”.
My owner, “Shinzo”, was once hospitalized for heart failure.
I don't want Sinzo to be admitted to hospital with worsening heart failure again.
So I'll tell you what all medical and care staffs in the community need to know.
Let’s use the heart failure handbook to share the patient's condition in the community.
Ask patients to write down their blood pressure, weight and symptoms in a handbook, and carry it when visiting outpatient clinic or care service facilities.
Medical and care staffs check the handbook not to miss the worsening heart failure. If there is weight gain, worsening breathlessness, or worsening swelling, whoever notice them should consult the family doctor.
The heart failure handbook has a contact note section, so share information in the community.
Let's work together in the community to prevent worsening heart failure and support patients in order to extend their healthy life expectancy.
All medical and care staff in the community are member of the heart failure team.
Let’s support the patients together!
Total time:1.38
Supplemental 3
