# Supplementary material for: Factors affecting the willingness of nursing care staffs for cooperation with heart failure care and the role of internet video education
Source: J Gen Fam Med. 2023 Nov 20;25(1):19–27. doi: 10.1002/jgf2.658 (PMC10792320; doi:10.1002/jgf2.658)
Supplement: Supplementary file 2 — Table S1. [file JGF2-25-19-s001.docx]

**Supplemental Table Study questionnaire.**

| **Survey of respondent’s demographics** | |
| --- | --- |
| Please tell us your age. | □20-29 y/o □30-39 y/o □40-49 y/o □50-59 y/o □60 or more y/o |
| Please tell us your gender. | □male □female |
| Please tell us your occupation. | Free comment ( ) |
| Please tell use your workplace facilities. | Free comment ( ) |
| **Questions about regional collaboration with HF care** | |
| Q1. How do you think about working together in the community to prevent HF? | □I’m not interested in cooperating with HF care □I’m interested in cooperating with HF care, but unwilling to do so □I’m interested in cooperating with HF care, and want to do so □I’m now cooperating with HF care, but I’m not confident to continue □I’m now cooperating with HF care and confident to continue |
| Q2. Are there opportunities to learn about HF? | □No opportunities at all □Few opportunities □Occasional opportunities  □Frequent opportunities |
| Q3. Would you like to participate in any opportunities to learn about HF? | □I don't want to participate □I want to participate □I want to participate very much: |
| Q4. Please tell us about any problems with cooperation with HF care. | Free comment ( ) |
| Q5. Please watch video 1. After watching video 1, did you understand the significance of working together in the community to prevent HF? | □Could not understand □Understood □Well understood |
| Q6. Please watch video 2. After watching video 2, did you understand the key points of observation in patients with HF (weight monitoring, observation of shortness of breath, presence of oedema) ? | □Could not understand □Understood □Well understood |
| Q7. Please watch video 3. After watching video 3, did you understand how to use the HF handbook to share the patient's condition in the community ? | □Could not understand □Understood □Well understood |
| Q8. After watching videos 1-3, do you think that the videos are a good way to learn about HF? | □Good □Good nor bad □Bad |
| Q9. After watching videos 1-3, how do you think about working together in the community to prevent HF? | □I’m not interested in cooperating with HF care □I’m interested in cooperating with HF care, but unwilling to do so □I’m interested in cooperating with HF care, and want to do so □I’m now cooperating with HF care, but I’m not confident to continue □I’m now cooperating with HF care and confident to continue |

y/o; years old, HF; heart failure.
